# Multifunctional Composites of Chiral Valine Derivative Schiff Base Cu(II) Complexes and TiO_2_

**DOI:** 10.3390/ijms16023955

**Published:** 2015-02-12

**Authors:** Yuki Takeshita, Kazuya Takakura, Takashiro Akitsu

**Affiliations:** Department of Chemistry, Faculty of Science, Tokyo University of Science, 1-3 Kagurazaka, Shinjuku-ku, Tokyo 162-8601, Japan; E-Mails: jb113697@ed.tus.ac.jp (Y.T.); metro.user@gmail.com (K.T.)

**Keywords:** Schiff base complexes, copper(II) complexes, UV–Vis spectra, TD-DFT, TiO_2_

## Abstract

We have prepared four new Cu(II) complexes containing valine moieties with imidazole ligands at the fourth coordination sites and examined their photo-induced reactions with TiO_2_ in order of understanding the reaction mechanisms. Under a nitrogen atmosphere, the intermolecular electron transfer reactions (essentially *supramolecular interactions*) of these systems, which resulted in the reduction of Cu(II) species to Cu(I) ones, occurred after UV light irradiation. In this study, we have investigated the conditions of the redox reactions in view of substituent effects of aldehyde moieties. The results of cyclic voltammetry (CV) on an rotating ring-disk electrode (RRDE) suggested that the substitution effects and redox potentials were correlated. Density functional theory (DFT) and time-dependent DFT (TD-DFT) calculations were also performed to simulate the UV–Vis and circular dichroism (CD) spectra; the results revealed a reasonably good correlation between the substituent effects and the highest occupied molecular orbitals and the lowest unoccupied molecular orbitals (HOMO-LUMO) gaps associated with the most intense transition bands. In addition, we summarized the substitution effects of Cu(II) complexes for their corresponding UV light-induced reactions.

## 1. Introduction

Inorganic nanoparticles such as TiO_2_ have been used in cosmetics for a long time because their optical absorption and scattering of UV light are remarkable. By controlling the nanoparticle size, it is typically possible to control the wavelength of light absorption and emission for many nanomaterials such as CdSe. Basic materials studies have been widely carried out in the field of cosmetology to search for materials and/or conditions to reduce UV light damage to the skin. Previous studies on inorganic oxides have mainly focused on chemical stability, UV absorption, and the uniform synthesis of nanoparticles [1,2,3,4,5,6,7,8,9,10,11].

Conversely, the absorption properties of metal complexes employed as UV light absorption agents to prevent UV damage can be easily controlled compared to their conventional organic counterparts. Indeed, organic dyes are not expected to absorb a wide wavelength range of UV light. In addition, biological and medical problems related to UV light, such as compatibility and incompatibility with proteins (polypeptide or amino acids), toxicity to the human body, and chemical stability of biological molecules like nucleic acids responsible for genetic information may also be important.

To take advantage of these inorganic nanoparticles and metal complexes such as UV absorbers, it is possible to control their absorption wavelengths and strongly absorbed UV light to react with TiO_2_ by compositing the metal complexes. And it can also be a dye. Therefore it is possible to develop UV protection dye complexes to improve conventional sunscreen material.

**Figure 1:**
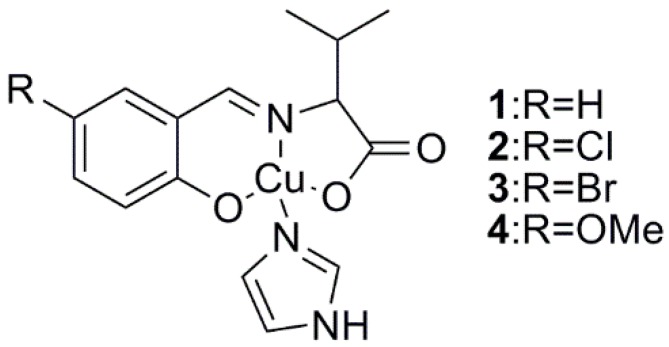
Molecular structures of Cu(II) complexes (**1**–**4**). Crystalline solvents (water molecules) are omitted for clarity.

In this manner, we have previously reported that some copper(II) complexes, especially Schiff base complexes with l-amino acid moieties, showed photo-induced electron transfer reactions with TiO_2_ after UV light irradiation [12,13,14,15,16,17,18,19]. Although limited spectral and electrochemical data could be obtained, the optimal reaction conditions along with the tuning of redox properties by molecular design are not yet fully understood. Herein, we prepared four new related Cu(II) complexes containing valine moieties (Figure 1) with imidazole ligands at the fourth coordination sites and examined their photo-induced reactions with TiO_2_ to understand their reaction mechanism.

## 2. Results and Discussion

### 2.1. Crystal Structures

**Figure 2:**
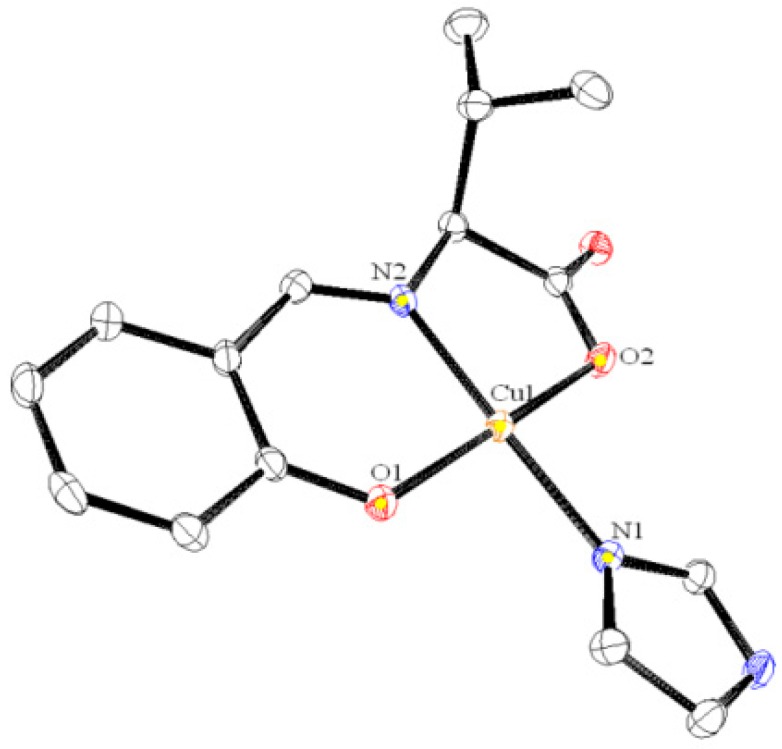
Crystal structure of **1** with selected atoms labeled. Hydrogen atoms are omitted for clarity.

**Figure 3:**
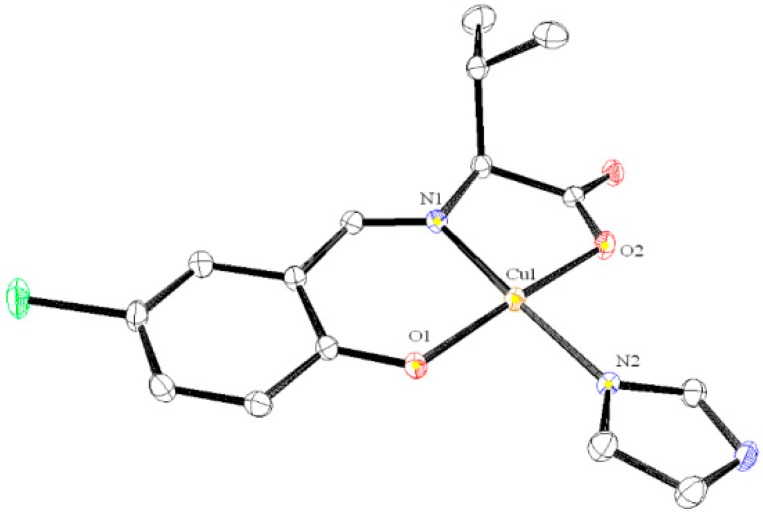
Crystal structures of **2** with selected atoms labeled. Hydrogen atoms are omitted for clarity.

**Figure 4:**
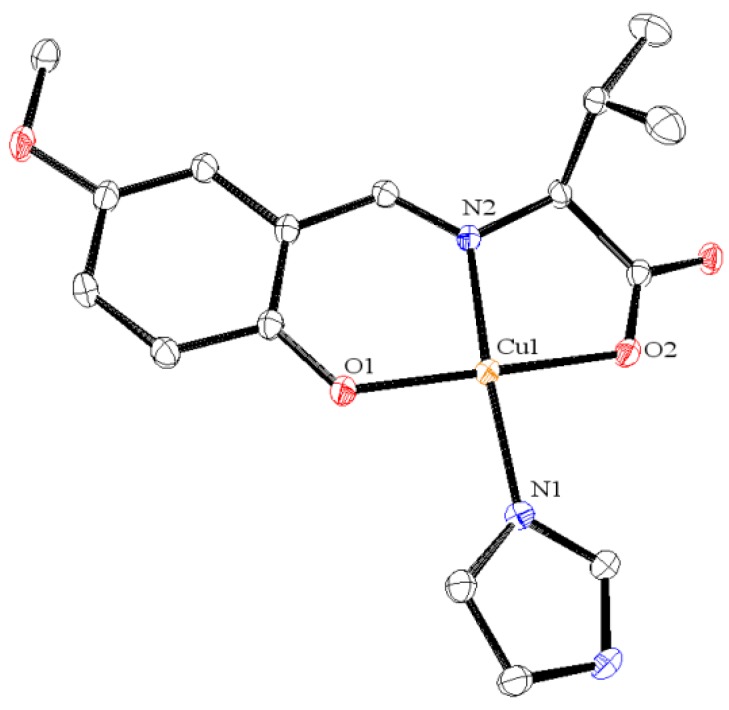
Crystal structures of **4** with selected atoms labeled. Hydrogen atoms are omitted for clarity.

The molecular structures of **1**, **2**, and **4** are depicted in Figure 2, Figure 3 and Figure 4, respectively. These compounds display slightly distorted square planar coordination geometries with umbrella conformations.

Selected bond distances (Å) and bond or torsion angles (°) for **1** are as follows: Cu1–O1 = 1.9167 (14), Cu1–O2 = 1.9755 (14), Cu1–N1 = 1.9585 (18), Cu1–N2 = 1.9244 (17), O1–Cu1–O2 = 171.27 (7), O1–Cu1–N1 = 91.61 (7), O1–Cu1–N2 = 94.11 (7), N1–Cu1–N2 = 172.37 (7).

Selected bond distances (Å) and bond or torsion angles (°) for **2** are as follows: Cu1–O1 = 1.916 (2), Cu1–O2 = 1.9705 (19), Cu1–N1 = 1.931 (2), Cu1–N2 = 1.958 (2), O1–Cu1–O2 = 170.94 (9), O1–Cu1–N1 = 94.00 (8), O1–Cu1–N2 = 91.61 (9), N1–Cu1–N2 = 172.65 (10).

Selected bond distances (Å) and bond or torsion angles (°) for **4** are as follows: Cu1–O1 = 1.919 (3), Cu–O2 = 1.981 (3), Cu1–N1 = 1.932 (3), Cu1–N2 = 1.959 (3), O1–Cu1–O2 = 172.29 (13), O1–Cu1–N1 = 93.56 (12), O1–Cu1–N2 = 92.23 (13), N1–Cu1–N2 = 171.46 (13).

Most of these values are within the normal ranges expected for the analogous Schiff base Cu(II) complexes [18,19]. The molecular packing in the solid state is essentially formed by weak van der Waals forces.

### 2.2. UV–Vis and CD Spectra

**Figure 5:**
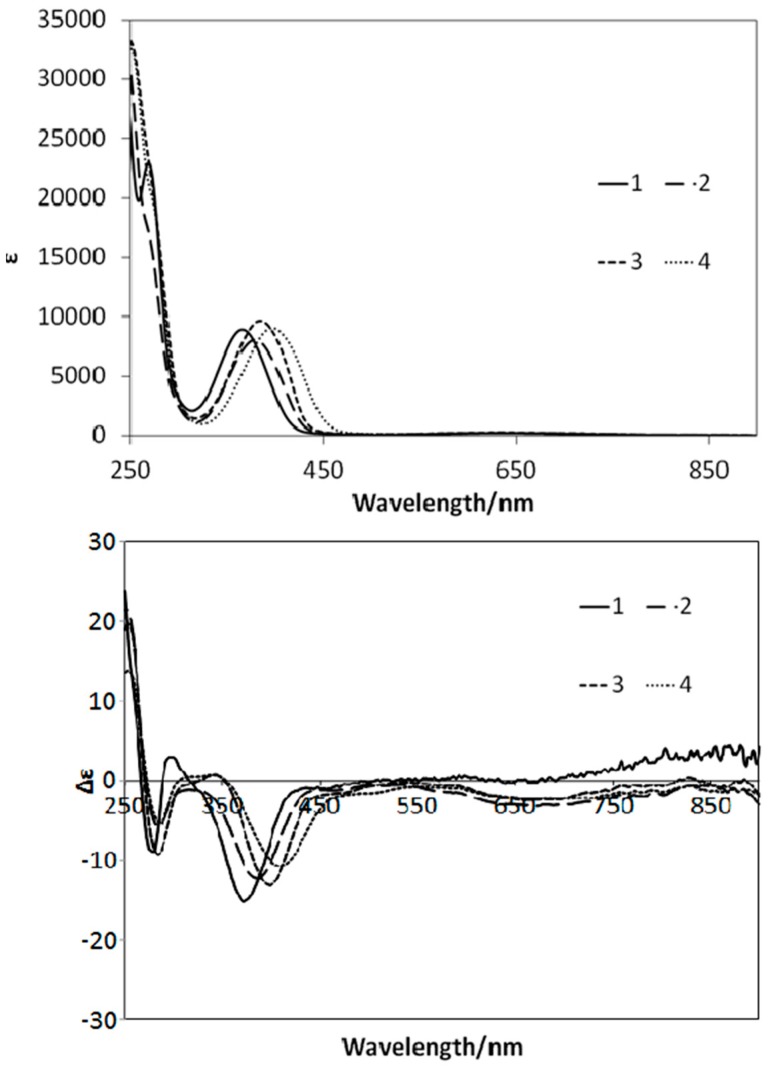
UV–Vis (**top**) and CD (**bottom**) spectra of **1**–**4** solutions in methanol.

Figure 5 depicts the UV–Vis and CD spectra of **1**–**4** for systematic comparison and discussion. All spectra predominantly exhibited intraligand π–π* and n–π* bands around 260–390 nm and relatively weak d–d bands around 500 nm due to the d^9^ electronic configuration of Cu(II) complexes. The assignment of the bands [20] in the experimental spectra is qualitatively in agreement with and supported by the computational results (see Section 2.4.). The electron-withdrawing halogen and methoxy substituent groups resulted in remarkable shifts in the UV–Vis peaks observed at 382 nm for **2**, 384 nm for **3**, and 400 nm for **4**.

### 2.3. Redox Potential

All complexes exhibited electrochemically reversible redox behavior based on the differences between the anode and cathode potentials. The CV results revealed that the redox potentials were *E* = 0.078, 0.014, 0.011, and −0.001 V for **1**, **2**, **3**, and **4**, respectively; among them, only **4** exhibited a negative redox potential (*E*). The four-coordinate valine derivative complexes in this study exhibited the same redox potential tendency (the order of *E* as –H > –Br > –OMe) as the analogous six-coordinated arginine derivative-Schiff base Cu(II) complexes (the order of *E* was also –H > –Br > –OMe) [21]. As known for highly electron-donating methoxy groups, the low *E* of **4** tends to stabilize the Cu(II) oxidation state.

### 2.4. Computational Results

**Figure 6:**
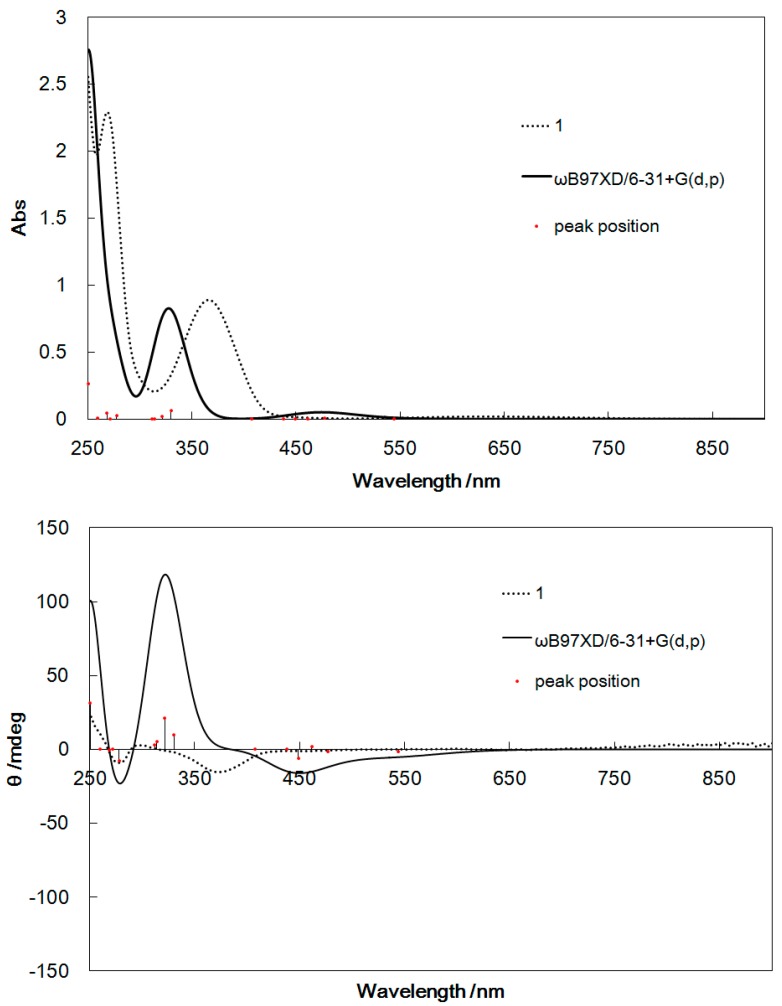
UV–Vis spectra (**top**) and CD spectra (**bottom**) of **1** calculated by TD-DFT and determined experimentally for comparison.

**Figure 7:**
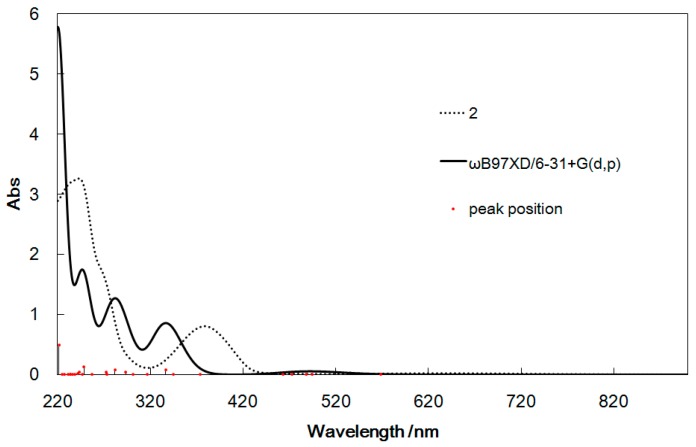

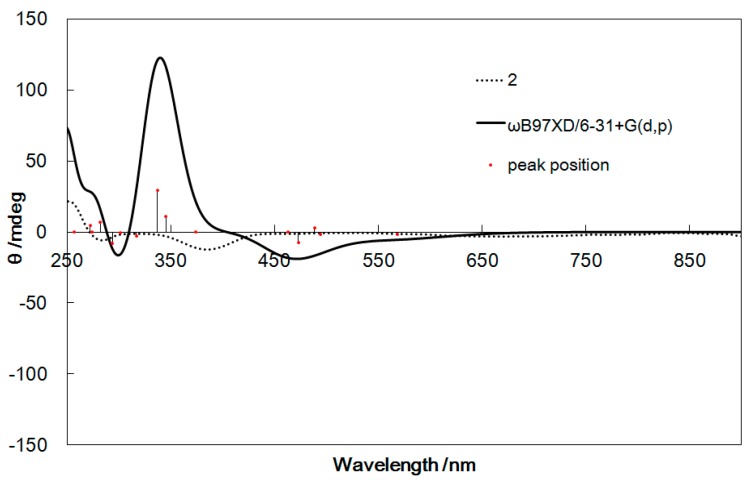
UV–Vis spectra (**top**) and CD spectra (**bottom**) of **2** calculated by TD-DFT and determined experimentally for comparison.

**Figure 8:**
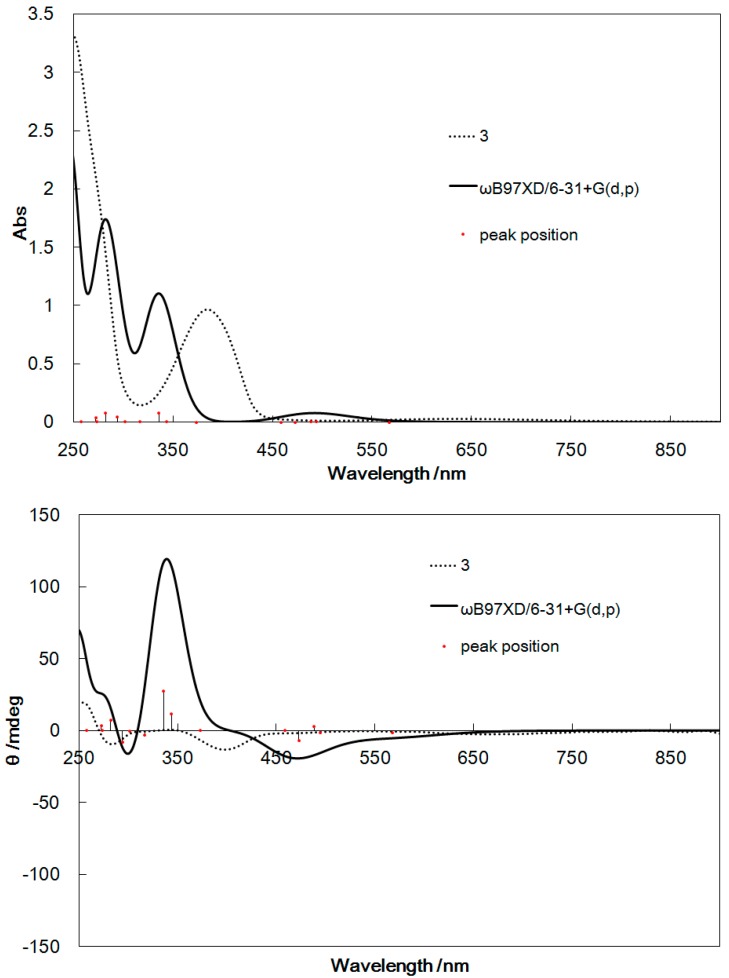
UV–Vis spectra (**top**) and CD spectra (**bottom**) of **3** calculated by TD-DFT and determined experimentally for comparison.

**Figure 9:**
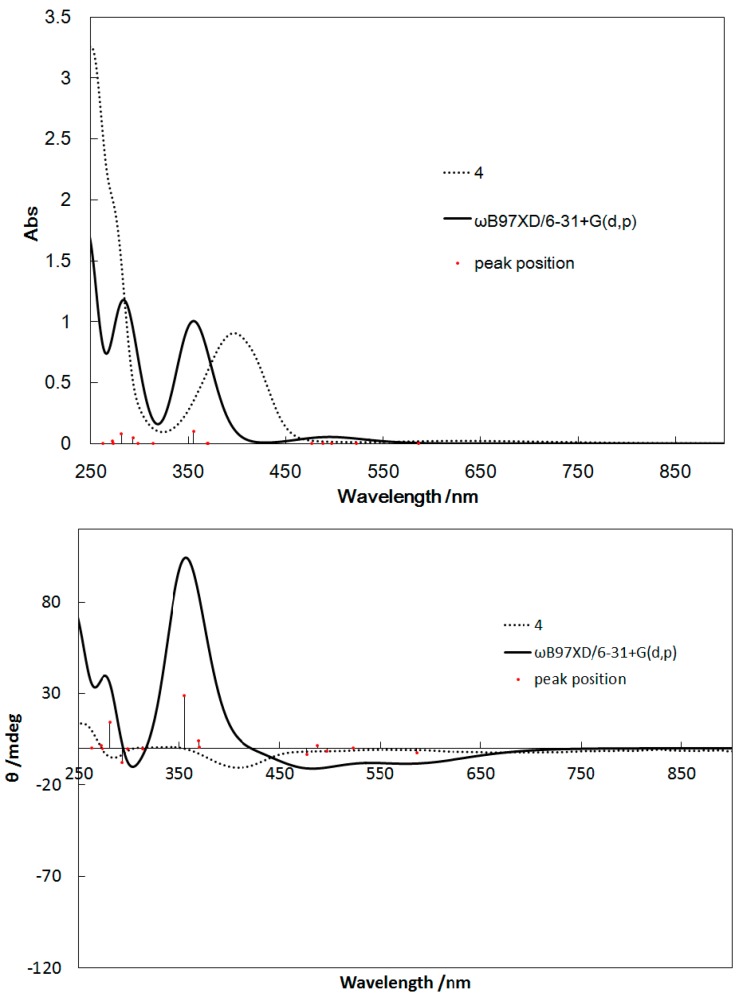
UV–Vis spectra (**top**) and CD spectra (**bottom**) of **4** calculated by TD-DFT and determined experimentally for comparison.

Figure 6, Figure 7, Figure 8, and Figure 9 show the UV–Vis and CD spectra for **1**–**4** simulated by Density functional theory (DFT) and time-dependent DFT (TD-DFT) calculations (whose 6-311+G(d,p) basis set provided similar results with ZINDO (Zerner’s Intermediate Neglect of Differential Overlap). The exchange-correlation function ωB97XD [22] was used, which included the long-range correction. The basis set used was 6-31+G(d,p), similar to general procedures. Considerable red shifts of the π–π* bands were observed for the simulated spectra of **1**–**4**, which may be attributed to the stabilization of the levels of the excited states by the polar methanol solvent. However, the band assignment was qualitatively reliable for all complexes.

### 2.5. UV Light-Induced Reactions

**Figure 10:**
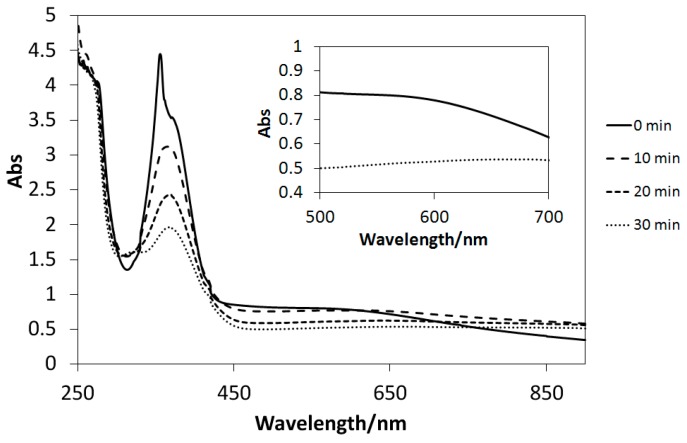
The UV–Vis spectral changes of **1** in the presence of TiO_2_ microparticles subjected to UV irradiation every 10 min.

**Figure 11:**
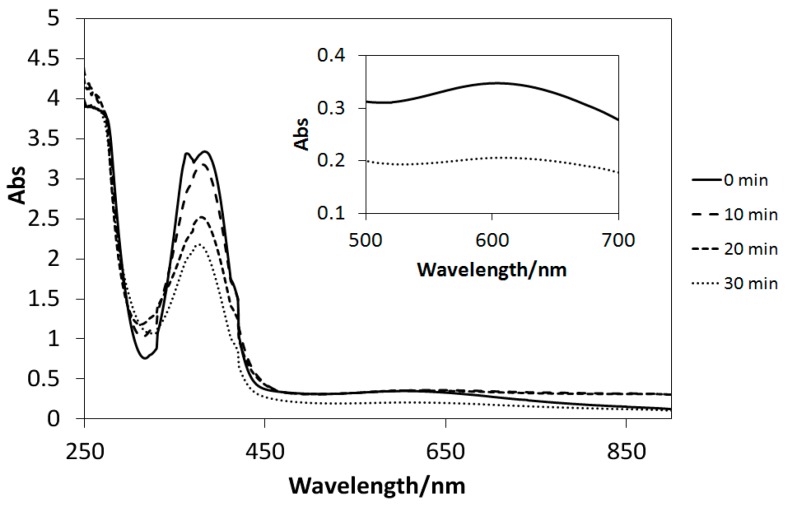
The UV–Vis spectral changes of **2** in the presence of TiO_2_ microparticles subjected to UV irradiation every 10 min.

**Figure 12:**
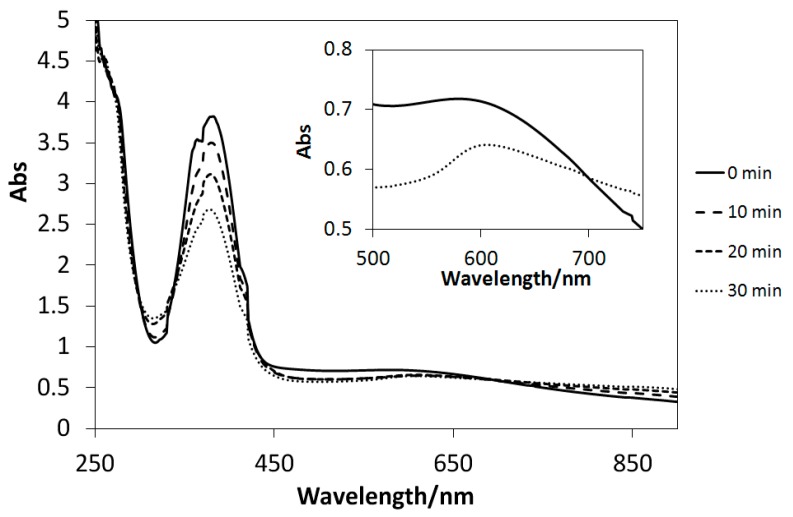
The UV–Vis spectral changes of **3** in the presence of TiO_2_ microparticles subjected to UV irradiation every 10 min.

**Figure 13:**
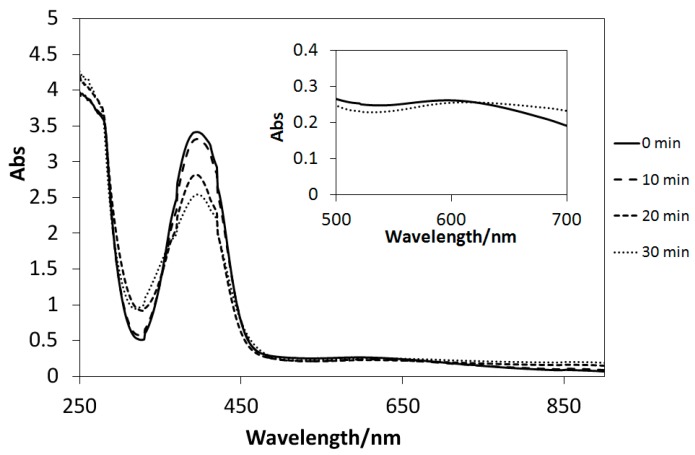
The UV–Vis spectral changes of **4** in the presence of TiO_2_ microparticles subjected to UV irradiation every 10 min.

The UV–Vis spectra of the solutions of Cu(II) complexes (**1**–**4**) dissolved in methanol exhibited only slight changes after UV light irradiation, similar to those of the analogous four-coordinated [20] and six-coordinated [21,23] Cu(II) complexes. Conversely, UV light-induced reactions could be induced in the presence of TiO_2_ microparticles (Figure 10, Figure 11, and Figure 12). Decreases in the intensities of the d–d bands around 600 nm suggested the reduction of d^9^ Cu(II) species (**1**–**3**) into d^10^ Cu(I) ones (the latter potentially incur charge transfer transitions by absorbing UV light). However, few spectral changes in the d–d bands are observed for Figure 13; this is due to negative *E* value of **4**, which causes it to react differently than the complexes with positive *E* values. Therefore, it is possible to control the reduction reactivity of the Cu(II) complexes by slight chemical modification of the ligands. Moreover, for the first time, the absorption intensities of the Cu(II) complexes around 360 nm (π–π* bands) were observed to decrease in suspensions of TiO_2_ particles; this may be ascribed to electron transfer reactions, which absorb extra UV light.

## 3. Experimental Section

### 3.1. General Procedures

Chemicals of the highest commercial grade available were purchased from Aldrich (St. Louis, MO, USA), Wako (Osaka, Japan), and TCI (Tokyo, Japan) and used as received without further purification.

### 3.2. Preparations

#### 3.2.1. Preparation of **1**

After reacting a methanol solution (50 mL) of l-valine (0.12 g, 1.0 mmol) and salicylaldehyde (0.12 g, 1.0 mmol) for 3 h at 333 K, Cu(CH_3_COO)_2_ (0.18 g, 1.0 mmol) was added. A solution of imidazole (0.13 g, 1.0 mmol) in methanol was then added and stirred for 2 h to yield a green precipitate by evaporation and filtration. The resulting crude compound was filtered and purified by recrystallization in methanol.

Yield 50.5%. Anal. Calc. for C_15_H_17_CuN_3_O_3_: C, 51.50; H, 4.61; N, 12.01%. Found: C, 51.35; H, 4.88; N, 11.98%. IR (infrared) (KBr, cm^−1^): 544 s, 626 w, 656 s, 738 m, 761 m, 852 m, 956 w, 1071 m, 1101 w, 1149 m, 1240 w, 1283 w, 1324 m, 1378 m, 1407 w, 1448 s, 1533 w, 1589 s, 1635 s (C=N), 2868 w, 2953 w, 3029 w, 3114 w, 3459 w. UV–Vis (diffuse reflectance): 606 nm (d–d), 354 nm (π–π*). ESR (electron spin resonance) (X-band, 1 mM methanol, 77 K): g = 2.007.

#### 3.2.2. Preparation of **2**

After reacting a methanol solution (50 mL) of l-valine (0.12 g, 1.0 mmol) and 5-chlorosalicylaldehyde (0.16 g, 1.0 mmol) for 3 h at 333 K, Cu(CH_3_COO)_2_ (0.18 g, 1.0 mmol) was added. A solution of imidazole (0.13 g, 1.0 mmol) in methanol was added and stirred for 2 h to yield a green precipitate by evaporation and filtration. The resulting crude compound was filtered and purified by recrystallization in methanol.

Yield 55.5%. Anal. Calc. for C_15_H_16_ClCuN_3_O_3_: C, 46.93; H, 4.07; N, 10.82%. Found: C, 46.76; H, 4.19; N, 11.03%. IR (KBr, cm^−1^): 555 s, 623 w, 657 s, 804 m, 826 s, 953 w, 1071 m, 1169 m, 1240 w, 1280 w, 1315 m, 1378 m, 1454 s, 1542 w, 1590 s, 1633 s (C=N), 2870 w, 2960 w, 3145 w, 3427 w. UV–Vis (diffuse reflectance): 608 nm (d–d), 354 nm (π–π*). ESR (X-band, 1 mM methanol, 77 K): g = 2.034.

#### 3.2.3. Preparation of **3**

After reacting a methanol solution (50 mL) of l-valine (0.12 g, 1.0 mmol) and 5-bromosalicylaldehyde (0.20 g, 1.0 mmol) for 3 h at 333 K, Cu(CH_3_COO)_2_ (0.18 g, 1.0 mmol) was added. A solution of imidazole (0.13 g, 1.0 mmol) in methanol was added and stirred for 2 h to yield a green precipitate by evaporation and filtration. The resulting crude compound was filtered and purified by recrystallization in methanol.

Yield 42.2%. Anal. Calc. for C_15_H_16_BrCuN_3_O_3_: C, 41.92; H, 3.75; N, 9.78%. Found: C, 41.99; H, 3.44; N, 9.05%. IR (KBr, cm^−1^): 550 s, 623 w, 656 s, 814 m, 826 s, 953 w, 1070 m, 1166 m, 1232 w, 1275w, 1312 m, 1371 m, 1453 s, 1540 w, 1589 s, 1633 s (C=N), 2863 w, 2958 w, 3149 w, 3430 w. UV–Vis (diffuse reflectance) 609 nm (d–d), 354 nm (π–π*). ESR (X-band, 1 mM methanol, 77 K): g = 2.028.

#### 3.2.4. Preparation of **4**

After reacting a methanol solution (50 mL) of l-valine (0.12 g, 1.0 mmol) and 2-hydroxy-5-metoxybenzaldehyde (0.15 g, 1.0 mmol) for 3 h at 333 K, Cu(CH_3_COO)_2_ (0.18 g, 1 mmol) was added. A solution of imidazole (0.13 g, 1.0 mmol) in methanol was added and stirred for 2 h to yield a green precipitate by evaporation and filtration. The resulting crude compound was filtered and purified by recrystallization in methanol.

Yield 44.8%. Anal. Calc. for C_16_H_19_CuN_3_O_4_: C, 50.45; H, 5.03; N, 11.03%. Found: C, 50.56; H, 4.88; N, 10.92%. IR (KBr, cm^−1^): 545 , 621 w, 650 s, 745 m, 776 m, 853 m, 956 w, 1070 m, 1101 w, 1155 m, 1278 w, 1289 w, 1320 m, 1390 m, 1405 w, 1450 s, 1523 w, 1590 s, 1632 s (C=N), 2890 w, 2952 w, 3021 w, 3110 w, 3470w. UV–Vis (diffuse reflectance): 612 nm (d–d), 358 nm (π–π*). ESR (X-band, 1 mM methanol, 77 K): g = 2.033.

### 3.3. Physical Measurements

Elemental analyses (C, H, N) were carried out with a Perkin-Elmer 2400II CHNS/O analyzer (Foster City, CA, USA) at Tokyo University of Science. Infrared spectra were recorded on a JASCO FT-IR 4200 plus spectrophotometer (JASCO Corporation, Tokyo, Japan) using KBr pellets in the range of 4000–400 cm^−1^ at 298 K. Absorption electronic spectra were measured on a JASCO V-570 spectrophotometer in the range of 900–200 nm at 298 K. Circular dichroism (CD) spectra were measured on a JASCO J-725 spectropolarimeter in the range of 900–200 nm at 298 K. X-band ESR spectra were measured with a JEOL JES-FA200 spectrometer (JEOL, Tokyo, Japan) at 77 K. Spectroelectrochemical measurements were carried out on BAS SEC2000-UV/CVIS and ALS2323 systems (BAS, Tokyo, Japan) with Ag/AgCl electrodes in aqueous solutions. Cyclic voltammetry (CV) measured with RRDE electrodes as Nafion films of ethanol solution of the complexes. The UV and visible light sources were Hayashi LA-310UV and LA-251Xe (Hayashi Watch Works, Tokyo, Japan), respectively, with visible (λ > 350 nm) and UV (λ < 350 nm) cut filters, respectively.

### 3.4. X-ray Crystallography

Deep-greenish prismatic single crystals of **1**, **2**, and **4** were glued on top of a glass fiber and coated with a thin layer of epoxy resin to measure the diffraction data. Intensity data were collected on a Bruker APEX2 CCD diffractometer with graphite monochromated Mo Kα radiation (λ = 0.71073 Å). Data analysis was carried out using the SAINT program package [24]. The structures were solved by direct methods with SHELXS-97 [25], expanded by Fourier techniques, and refined by full-matrix least-squares methods based on *F*^2^ using the program SHELXL-97. An empirical absorption correction was applied by the program SADABS [26]. All non-hydrogen atoms were readily located and refined by anisotropic thermal parameters. All hydrogen atoms were located at geometrically calculated positions and refined using riding models. Unfortunately, single crystals of **3** could not be obtained.

#### 3.4.1. Crystallographic Data for **1**

C_15_H_17_CuN_3_O_3_, crystal size 0.28 mm × 0.21 mm × 0.19 mm, *M*w = 350.86, tetragonal, space group *P*2_1_ (#4), *a* = 5.3136(7) Å, *b* = 8.8313(11) Å, *c* = 16.190(2) Å, *V* = 759.70(17) Å^3^, *Z* = 2, *D*_calc_ = 1.534 mg/m^3^, *F*(000) = 394, *R*_1_ = 0.0221, *wR*_2_ = 0.0530 (3514 reflections), *S* = 0.625, Flack parameter = 0.020(10) (where *R*_1_ = ∑ ||*F*_o_| − |*F*_c_||/∑ |F_o_|, *R*_w_ = (∑ *w*(|*F*_o_| − |*F*_c_|)^2^/∑ *w*|*F*_o_|^2^)^1/2^, *w* = 1/(σ^2^(*F*_o_) + (0.1*P*)^2^), *P* = (*F*_o2_ + 2*F*_c2_)/3).

#### 3.4.2. Crystallographic Data for **2**

C_15_H_16_ClCuN_3_O_3_, crystal size 0.14 mm × 0.13 mm × 0.09 mm, *M*w = 385.30, tetragonal, space group *P*2_1_2_1_2_1_ *(#*19*)*, *a* = 5.2964(16) Å, *b* = 8.759(3) Å, *c* = 34.915(10) Å, *V* = 1619.8(8) Å^3^, *Z* = 4, *D*_calc_ = 1.580 mg/m^3^, F(000) = 788, *R*_1_ = 0.0292, *wR*_2_ = 0.0909 (3287 reflections), *S* = 0.625, Flack parameter = 0.028(13). (where *R*_1_ = ∑ ||*F*_o_| − |*F*_c_||/∑ |F_o_|, *R*_w_ = (∑ *w*(|*F*_o_| − |F_c_|)^2^/∑ *w*|*F*_o_|^2^)^1/2^, *w* = 1/(σ^2^(*F*_o_) + (0.1*P*)^2^), *P* = (*F*_o2_ + 2*F*_c2_)/3).

#### 3.4.3. Crystallographic Data for **4**

C_16_H_19_CuN_3_O_4_, crystal size 0.18 mm × 0.15 mm × 0.12 mm, *M*w = 380.88, tetragonal, space group *P*2_1_ (#4), *a* = 5.3748(5) Å, *b* = 8.8801(9) Å, *c* = 17.2714(17) Å, *V* = 824.34(14) Å^3^, *Z* = 2, *D*_calc_ = 1.350 mg/m^3^, *F*(000) = 394, *R*_1_ = 0.0271, *wR*_2_ = 0.0745 (4626 reflections), *S* = 0.625, Flack parameter = 0.006(7) (where *R*_1_ = ∑ ||*F*_o_| − |*F*_c_||/∑ |*F*_o_|, *R*_w_ = (∑ *w*(|*F*_o_| − |*F*_c_|)^2^/∑ *w*|*F*_o_|^2^)^1/2^, *w* = 1/(σ^2^(*F*_o_) + (0.1*P*)^2^), *P* = (*F*_o2_ + 2*F*_c2_)/3).

### 3.5. Computational Methods

All calculations were performed using the Gaussian 09W software Revision D.01 (Gaussian, Inc., Wallingford, CT, USA) [27]. The vertical excitation energy was calculated using the TD-DFT method based on the singlet ground state geometry. The exchange functional, the correlation functional, and the basis set were ωB97XD/6-31+G(d,p). With the exception of **3**, crystal structure data were used as the initial structures.

## 4. Conclusions

We have synthesized **1**–**4** and investigated their photo-induced reactions with TiO_2_. The Cu(II) complexes alone showed no changes after UV light irradiation. However, composite systems (Cu(II) complexes + TiO_2_) showed characteristic intermolecular electron transfer reactions that depended on the ligands.

Composite systems containing **1**, **2**, and **3** showed decreases in d–d and π–π* band intensities, while the composite system of **4** only exhibited a decrease in the π–π* band intensity (no change in the d–d band). The CV and redox potential results were in accordance with the decreases in the absorption intensities of the d–d bands. Only **4** exhibited a negative redox potential, and the intensity of its d–d band decreased less than those of **1**, **2**, and **3**, which exhibited positive redox potentials. Complex **4** contains a methoxy group, which acts as a strong electron-donating agent; therefore, **4** tends to stabilize the Cu(II) state. This lowers the redox potential, suppressing the decrease in the intensity of the d–d band.

We have found that combining TiO_2_ and suitable metal complexes results in the occurrence of electron transfer reactions, which absorb extra UV light. Moreover, as demonstrated by the negative redox potential of the composite of **4**, photo-induced electron transfer can be controlled by the structures of Cu(II) complexes. Therefore, by taking advantage of molecular design, more functional applications (UV-color change and stable chemical species) of metal complexes can be expected.

## Supplementary Information

CCDC 1036495, 1036496, and 1036495 contain the supplementary crystallographic data for **1**, **2**, and **4**, respectively. These data can be obtained free of charge via http://www.ccdc.cam.ac.uk/conts/retrieving.html, or from the Cambridge Crystallographic Data Centre, 12 Union Road, Cambridge CB2 1EZ, UK; Fax: (+44) 1223-336-033; or E-Mail: deposit@ccdc.cam.ac.uk.
